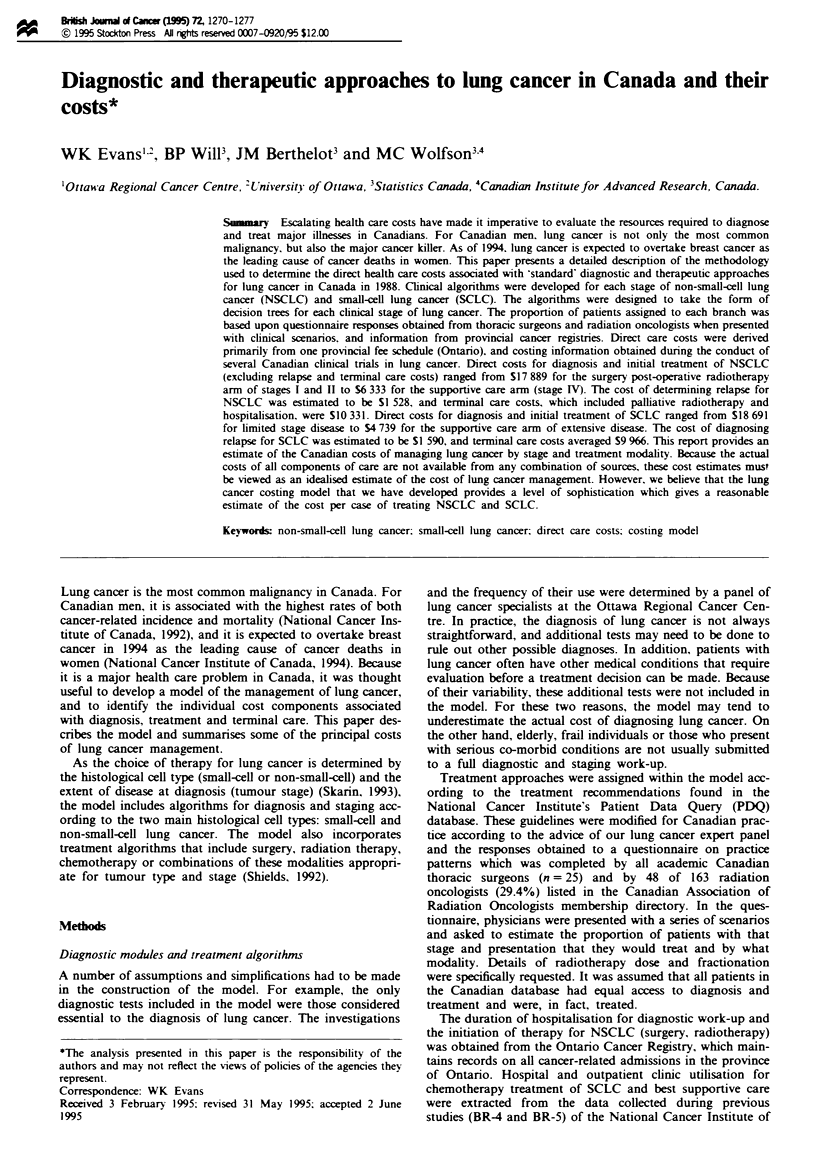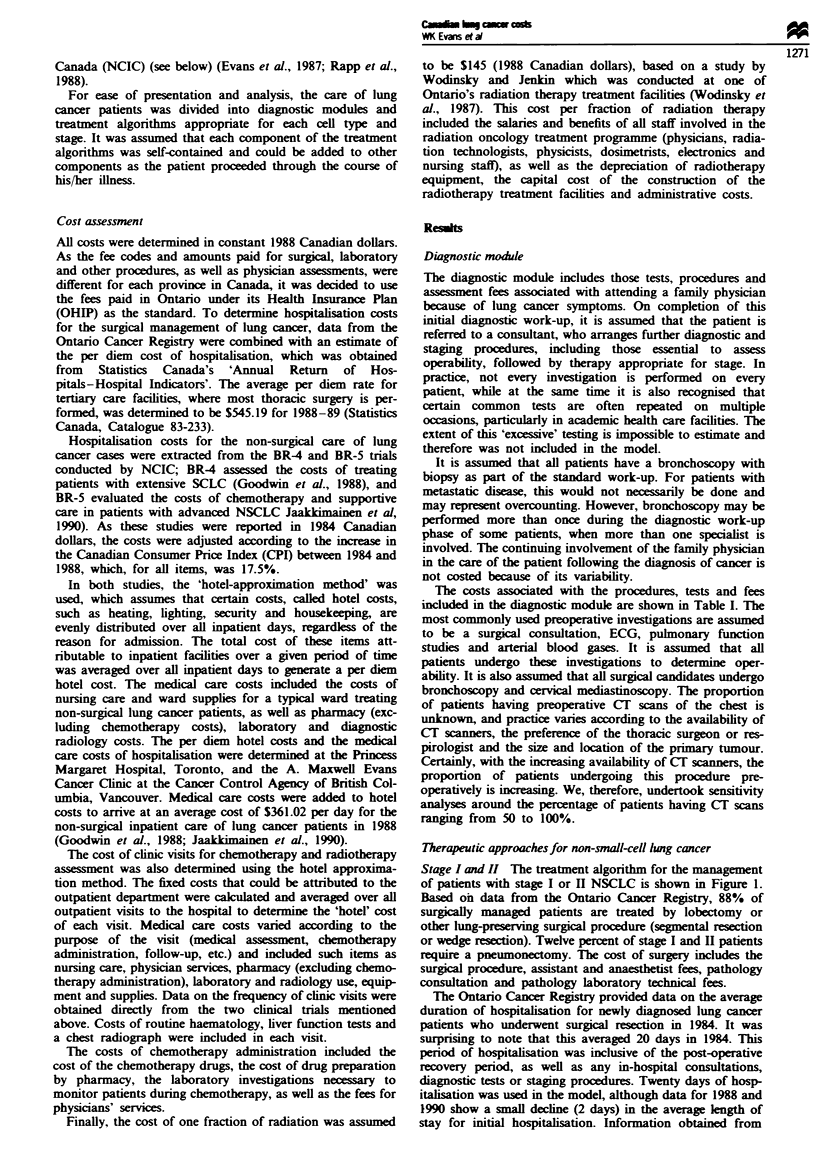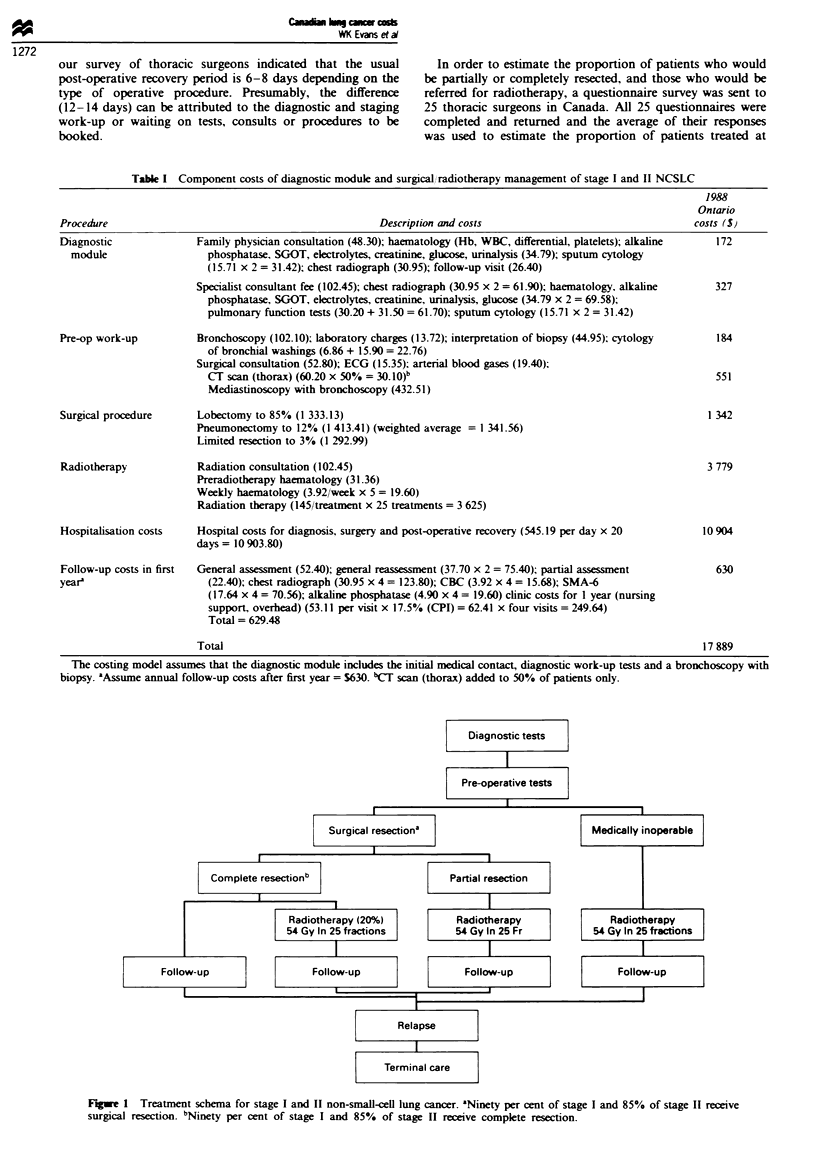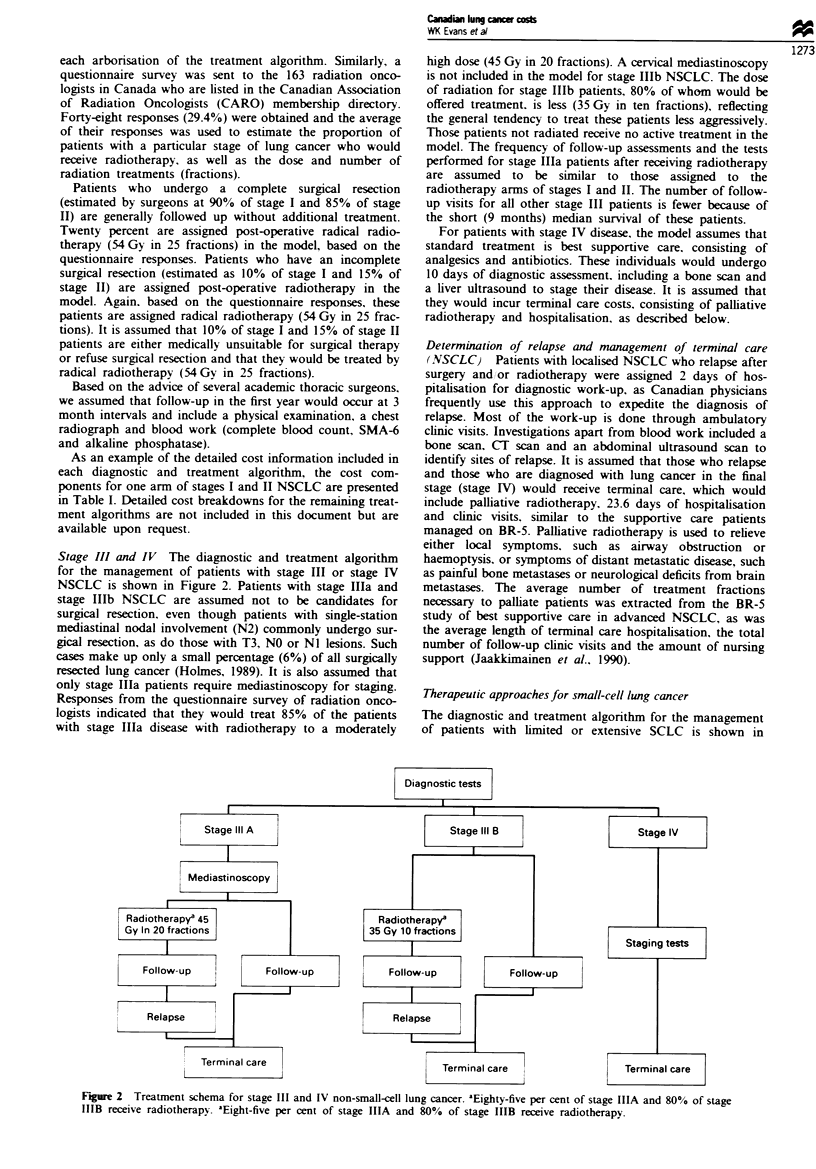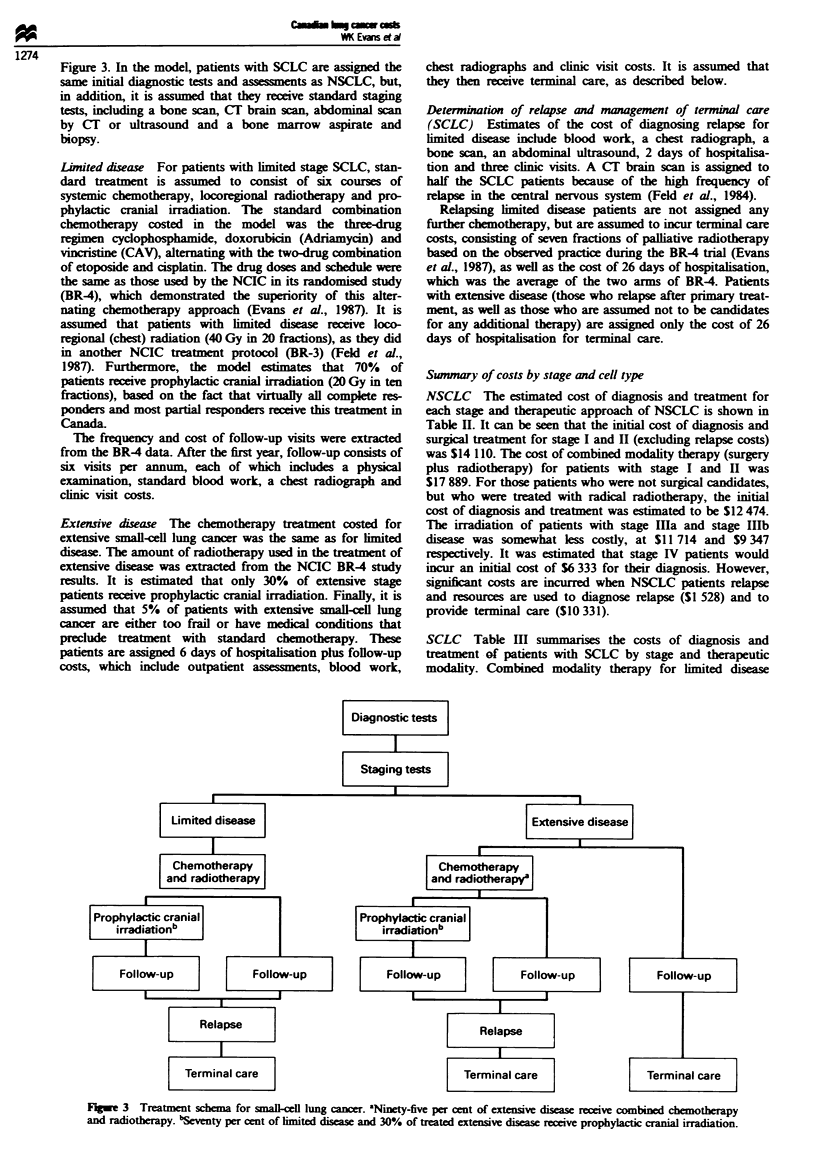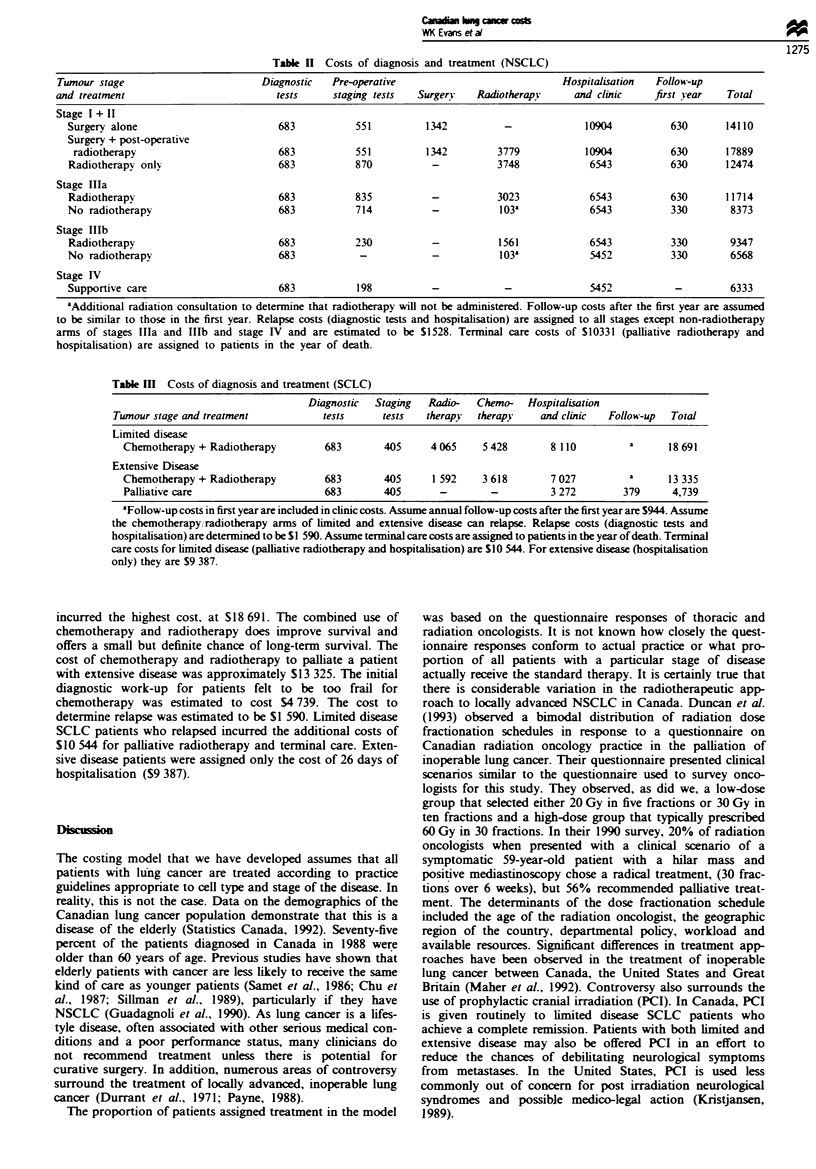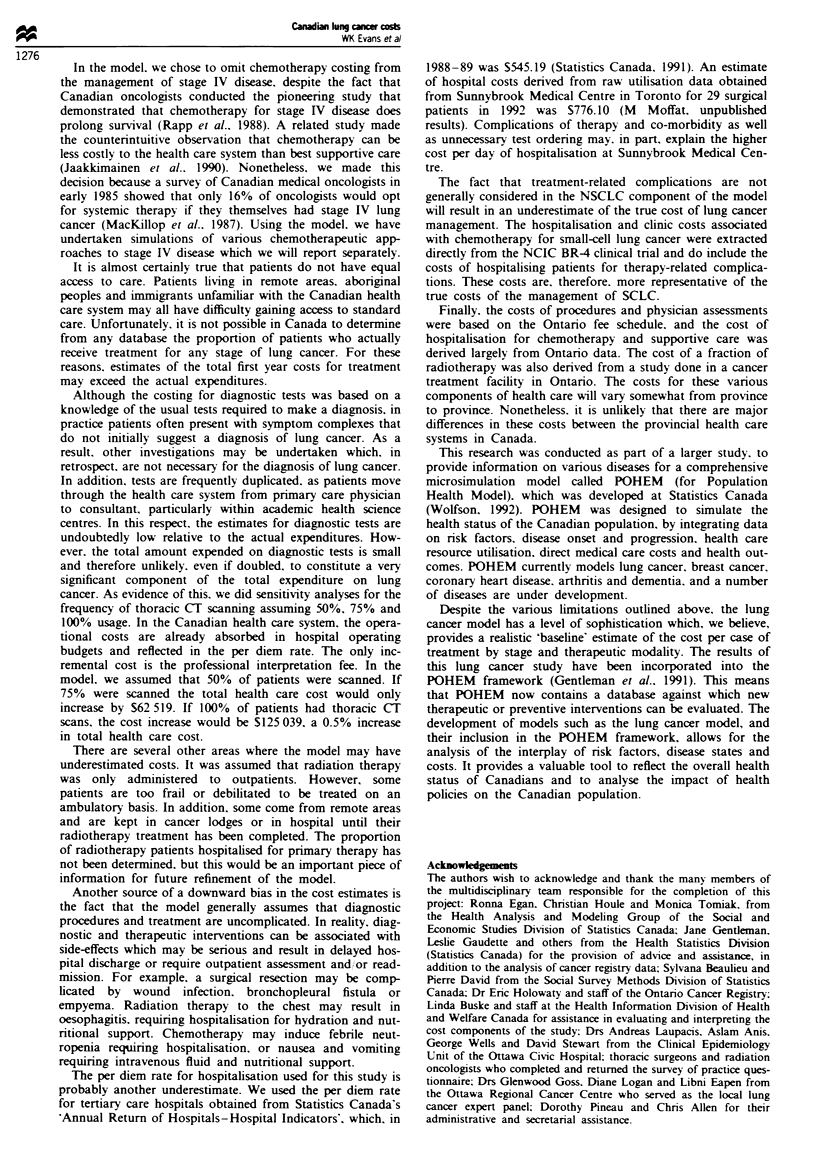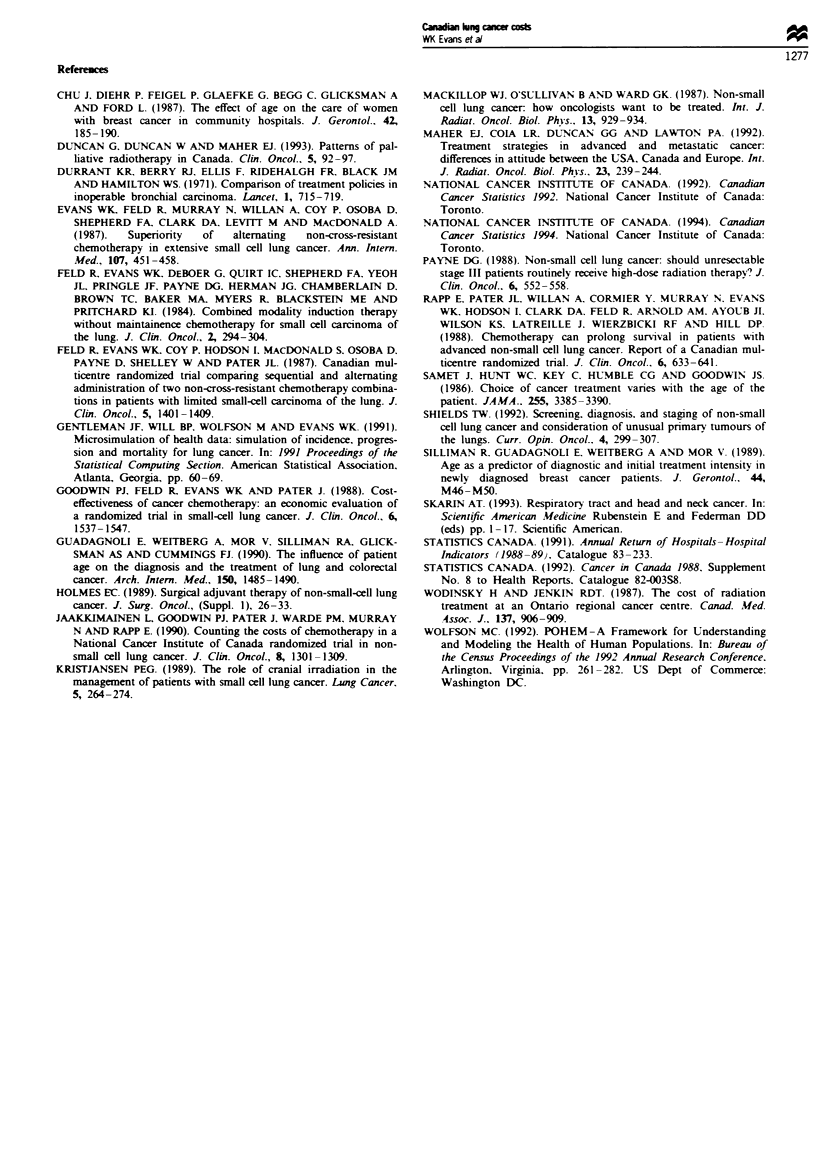# Diagnostic and therapeutic approaches to lung cancer in Canada and their costs.

**DOI:** 10.1038/bjc.1995.499

**Published:** 1995-11

**Authors:** W. K. Evans, B. P. Will, J. M. Berthelot, M. C. Wolfson

**Affiliations:** Ottawa Regional Cancer Centre, Canada.

## Abstract

Escalating health care costs have made it imperative to evaluate the resources required to diagnose and treat major illnesses in Canadians. For Canadian men, lung cancer is not only the most common malignancy, but also the major cancer killer. As of 1994, lung cancer is expected to overtake breast cancer as the leading cause of cancer deaths in women. This paper presents a detailed description of the methodology used to determine the direct health care costs associated with 'standard' diagnostic and therapeutic approaches for lung cancer in Canada in 1988. Clinical algorithms were developed for each stage of non-small-cell lung cancer (NSCLC) and small-cell lung cancer (SCLC). The algorithms were designed to take the form of decision trees for each clinical stage of lung cancer. The proportion of patients assigned to each branch was based upon questionnaire responses obtained from thoracic surgeons and radiation oncologists when presented with clinical scenarios, and information from provincial cancer registries. Direct care costs were derived primarily from one provincial fee schedule (Ontario), and costing information obtained during the conduct of several Canadian clinical trials in lung cancer. Direct costs for diagnosis and initial treatment of NSCLC (excluding relapse and terminal care costs) ranged from $17,889 for the surgery/post-operative radiotherapy arm of stages I and II to $6,333 for the supportive care arm (stage IV). The cost of determining relapse for NSCLC was estimated to be $1,528, and terminal care costs, which included palliative radiotherapy and hospitalisation, were $10,331. Direct costs for diagnosis and initial treatment of SCLC ranged from $18,691 for limited stage disease to $4,739 for the supportive care arm of extensive disease. The cost of diagnosing relapse for SCLC was estimated to be $1,590, and terminal care costs averaged $9,966. This report provides an estimate of the Canadian costs of managing lung cancer by stage and treatment modality. Because the actual costs of all components of care are not available from any combination of sources, these cost estimates must be viewed as an idealised estimate of the cost of lung cancer management. However, we believe that the lung cancer costing model that we have developed provides a level of sophistication which gives a reasonable estimate of the cost per case of treating NSCLC and SCLC.


British Journal of Cancer (1995) 72, 1270-1277

© 1995 Stockton Press All rights reserved 0007-0920/95 $12.00

### Diagnostic and therapeutic approaches to lung cancer in Canada and their costs*

WK Evans[1,2], BP Will[3], JM Berthelot[3] and MC Wolfson[3,4]

[1]*Ottawa Regional Cancer Centre,* [2]*University of Ottawa,* [3]*Statistics Canada,* [4]*Canadian Institute for Advanced Research, Canada.*

**Summary** Escalating health care costs have made it imperative to evaluate the resources required to diagnose and treat major illnesses in Canadians. For Canadian men, lung cancer is not only the most common malignancy, but also the major cancer killer. As of 1994, lung cancer is expected to overtake breast cancer as the leading cause of cancer deaths in women. This paper presents a detailed description of the methodology used to determine the direct health care costs associated with 'standard' diagnostic and therapeutic approaches for lung cancer in Canada in 1988. Clinical algorithms were developed for each stage of non-small-cell lung cancer (NSCLC) and small-cell lung cancer (SCLC). The algorithms were designed to take the form of decision trees for each clinical stage of lung cancer. The proportion of patients assigned to each branch was based upon questionnaire responses obtained from thoracic surgeons and radiation oncologists when presented with clinical scenarios, and information from provincial cancer registries. Direct care costs were derived primarily from one provincial fee schedule (Ontario), and costing information obtained during the conduct of several Canadian clinical trials in lung cancer. Direct costs for diagnosis and initial treatment of NSCLC (excluding relapse and terminal care costs) ranged from $17 889 for the surgery post-operative radiotherapy arm of stages I and II to $6 333 for the supportive care arm (stage IV). The cost of determining relapse for NSCLC was estimated to be $1 528, and terminal care costs, which included palliative radiotherapy and hospitalisation, were $10 331. Direct costs for diagnosis and initial treatment of SCLC ranged from $18 691 for limited stage disease to $4 739 for the supportive care arm of extensive disease. The cost of diagnosing relapse for SCLC was estimated to be $1 590, and terminal care costs averaged $9 966. This report provides an estimate of the Canadian costs of managing lung cancer by stage and treatment modality. Because the actual costs of all components of care are not available from any combination of sources, these cost estimates must be viewed as an idealised estimate of the cost of lung cancer management. However, we believe that the lung cancer costing model that we have developed provides a level of sophistication which gives a reasonable estimate of the cost per case of treating NSCLC and SCLC.

**Keywords:** non-small-cell lung cancer; small-cell lung cancer; direct care costs; costing model

Lung cancer is the most common malignancy in Canada. For Canadian men, it is associated with the highest rates of both cancer-related incidence and mortality (National Cancer Institute of Canada, 1992), and it is expected to overtake breast cancer in 1994 as the leading cause of cancer deaths in women (National Cancer Institute of Canada, 1994). Because it is a major health care problem in Canada, it was thought useful to develop a model of the management of lung cancer, and to identify the individual cost components associated with diagnosis, treatment and terminal care. This paper describes the model and summarises some of the principal costs of lung cancer management.

As the choice of therapy for lung cancer is determined by the histological cell type (small-cell or non-small-cell) and the extent of disease at diagnosis (tumour stage) (Skarin, 1993), the model includes algorithms for diagnosis and staging according to the two main histological cell types: small-cell and non-small-cell lung cancer. The model also incorporates treatment algorithms that include surgery, radiation therapy, chemotherapy or combinations of these modalities appropriate for tumour type and stage (Shields, 1992).

### Methods

### *Diagnostic modules and treatment algorithms*

A number of assumptions and simplifications had to be made in the construction of the model. For example, the only diagnostic tests included in the model were those considered essential to the diagnosis of lung cancer. The investigations and the frequency of their use were determined by a panel of lung cancer specialists at the Ottawa Regional Cancer Centre. In practice, the diagnosis of lung cancer is not always straightforward, and additional tests may need to be done to rule out other possible diagnoses. In addition, patients with lung cancer often have other medical conditions that require evaluation before a treatment decision can be made. Because of their variability, these additional tests were not included in the model. For these two reasons, the model may tend to underestimate the actual cost of diagnosing lung cancer. On the other hand, elderly, frail individuals or those who present with serious co-morbid conditions are not usually submitted to a full diagnostic and staging work-up.

Treatment approaches were assigned within the model according to the treatment recommendations found in the National Cancer Institute's Patient Data Query (PDQ) database. These guidelines were modified for Canadian practice according to the advice of our lung cancer expert panel and the responses obtained to a questionnaire on practice patterns which was completed by all academic Canadian thoracic surgeons ($n = 25$) and by 48 of 163 radiation oncologists (29.4%) listed in the Canadian Association of Radiation Oncologists membership directory. In the questionnaire, physicians were presented with a series of scenarios and asked to estimate the proportion of patients with that stage and presentation that they would treat and by what modality. Details of radiotherapy dose and fractionation were specifically requested. It was assumed that all patients in the Canadian database had equal access to diagnosis and treatment and were, in fact, treated.

The duration of hospitalisation for diagnostic work-up and the initiation of therapy for NSCLC (surgery, radiotherapy) was obtained from the Ontario Cancer Registry, which maintains records on all cancer-related admissions in the province of Ontario. Hospital and outpatient clinic utilisation for chemotherapy treatment of SCLC and best supportive care were extracted from the data collected during previous studies (BR-4 and BR-5) of the National Cancer Institute of

*The analysis presented in this paper is the responsibility of the authors and may not reflect the views of policies of the agencies they represent.

Correspondence: WK Evans
Received 3 February 1995; revised 31 May 1995; accepted 2 June 1995

Canada (NCIC) (see below) (Evans et al., 1987; Rapp et al., 1988).

For ease of presentation and analysis, the care of lung cancer patients was divided into diagnostic modules and treatment algorithms appropriate for each cell type and stage. It was assumed that each component of the treatment algorithms was self-contained and could be added to other components as the patient proceeded through the course of his/her illness.

*Cost assessment*

All costs were determined in constant 1988 Canadian dollars. As the fee codes and amounts paid for surgical, laboratory and other procedures, as well as physician assessments, were different for each province in Canada, it was decided to use the fees paid in Ontario under its Health Insurance Plan (OHIP) as the standard. To determine hospitalisation costs for the surgical management of lung cancer, data from the Ontario Cancer Registry were combined with an estimate of the per diem cost of hospitalisation, which was obtained from Statistics Canada's 'Annual Return of Hospitals-Hospital Indicators'. The average per diem rate for tertiary care facilities, where most thoracic surgery is performed, was determined to be $545.19 for 1988-89 (Statistics Canada, Catalogue 83-233).

Hospitalisation costs for the non-surgical care of lung cancer cases were extracted from the BR-4 and BR-5 trials conducted by NCIC; BR-4 assessed the costs of treating patients with extensive SCLC (Goodwin et al., 1988), and BR-5 evaluated the costs of chemotherapy and supportive care in patients with advanced NSCLC Jaakkimainen et al, 1990). As these studies were reported in 1984 Canadian dollars, the costs were adjusted according to the increase in the Canadian Consumer Price Index (CPI) between 1984 and 1988, which, for all items, was 17.5%.

In both studies, the 'hotel-approximation method' was used, which assumes that certain costs, called hotel costs, such as heating, lighting, security and housekeeping, are evenly distributed over all inpatient days, regardless of the reason for admission. The total cost of these items attributable to inpatient facilities over a given period of time was averaged over all inpatient days to generate a per diem hotel cost. The medical care costs included the costs of nursing care and ward supplies for a typical ward treating non-surgical lung cancer patients, as well as pharmacy (excluding chemotherapy costs), laboratory and diagnostic radiology costs. The per diem hotel costs and the medical care costs of hospitalisation were determined at the Princess Margaret Hospital, Toronto, and the A. Maxwell Evans Cancer Clinic at the Cancer Control Agency of British Columbia, Vancouver. Medical care costs were added to hotel costs to arrive at an average cost of $361.02 per day for the non-surgical inpatient care of lung cancer patients in 1988 (Goodwin et al., 1988; Jaakkimainen et al., 1990).

The cost of clinic visits for chemotherapy and radiotherapy assessment was also determined using the hotel approximation method. The fixed costs that could be attributed to the outpatient department were calculated and averaged over all outpatient visits to the hospital to determine the 'hotel' cost of each visit. Medical care costs varied according to the purpose of the visit (medical assessment, chemotherapy administration, follow-up, etc.) and included such items as nursing care, physician services, pharmacy (excluding chemotherapy administration), laboratory and radiology use, equipment and supplies. Data on the frequency of clinic visits were obtained directly from the two clinical trials mentioned above. Costs of routine haematology, liver function tests and a chest radiograph were included in each visit.

The costs of chemotherapy administration included the cost of the chemotherapy drugs, the cost of drug preparation by pharmacy, the laboratory investigations necessary to monitor patients during chemotherapy, as well as the fees for physicians' services.

Finally, the cost of one fraction of radiation was assumed to be $145 (1988 Canadian dollars), based on a study by Wodinsky and Jenkin which was conducted at one of Ontario's radiation therapy treatment facilities (Wodinsky et al., 1987). This cost per fraction of radiation therapy included the salaries and benefits of all staff involved in the radiation oncology treatment programme (physicians, radiation technologists, physicists, dosimetrists, electronics and nursing staff), as well as the depreciation of radiotherapy equipment, the capital cost of the construction of the radiotherapy treatment facilities and administrative costs.

### Results

*Diagnostic module*

The diagnostic module includes those tests, procedures and assessment fees associated with attending a family physician because of lung cancer symptoms. On completion of this initial diagnostic work-up, it is assumed that the patient is referred to a consultant, who arranges further diagnostic and staging procedures, including those essential to assess operability, followed by therapy appropriate for stage. In practice, not every investigation is performed on every patient, while at the same time it is also recognised that certain common tests are often repeated on multiple occasions, particularly in academic health care facilities. The extent of this 'excessive' testing is impossible to estimate and therefore was not included in the model.

It is assumed that all patients have a bronchoscopy with biopsy as part of the standard work-up. For patients with metastatic disease, this would not necessarily be done and may represent overcounting. However, bronchoscopy may be performed more than once during the diagnostic work-up phase of some patients, when more than one specialist is involved. The continuing involvement of the family physician in the care of the patient following the diagnosis of cancer is not costed because of its variability.

The costs associated with the procedures, tests and fees included in the diagnostic module are shown in Table I. The most commonly used preoperative investigations are assumed to be a surgical consultation, ECG, pulmonary function studies and arterial blood gases. It is assumed that all patients undergo these investigations to determine operability. It is also assumed that all surgical candidates undergo bronchoscopy and cervical mediastinoscopy. The proportion of patients having preoperative CT scans of the chest is unknown, and practice varies according to the availability of CT scanners, the preference of the thoracic surgeon or respirologist and the size and location of the primary tumour. Certainly, with the increasing availability of CT scanners, the proportion of patients undergoing this procedure preoperatively is increasing. We, therefore, undertook sensitivity analyses around the percentage of patients having CT scans ranging from 50 to 100%.

*Therapeutic approaches for non-small-cell lung cancer*

*Stage I and II* The treatment algorithm for the management of patients with stage I or II NSCLC is shown in Figure 1. Based on data from the Ontario Cancer Registry, 88% of surgically managed patients are treated by lobectomy or other lung-preserving surgical procedure (segmental resection or wedge resection). Twelve percent of stage I and II patients require a pneumonectomy. The cost of surgery includes the surgical procedure, assistant and anaesthetist fees, pathology consultation and pathology laboratory technical fees.

The Ontario Cancer Registry provided data on the average duration of hospitalisation for newly diagnosed lung cancer patients who underwent surgical resection in 1984. It was surprising to note that this averaged 20 days in 1984. This period of hospitalisation was inclusive of the post-operative recovery period, as well as any in-hospital consultations, diagnostic tests or staging procedures. Twenty days of hospitalisation was used in the model, although data for 1988 and 1990 show a small decline (2 days) in the average length of stay for initial hospitalisation. Information obtained from our survey of thoracic surgeons indicated that the usual post-operative recovery period is 6–8 days depending on the type of operative procedure. Presumably, the difference (12–14 days) can be attributed to the diagnostic and staging work-up or waiting on tests, consults or procedures to be booked.

In order to estimate the proportion of patients who would be partially or completely resected, and those who would be referred for radiotherapy, a questionnaire survey was sent to 25 thoracic surgeons in Canada. All 25 questionnaires were completed and returned and the average of their responses was used to estimate the proportion of patients treated at

**Table I** Component costs of diagnostic module and surgical/radiotherapy management of stage I and II NCSLC

| *Procedure* | *Description and costs* | *1988 Ontario costs ($)* |
|---|---|---|
| Diagnostic module | Family physician consultation (48.30); haematology (Hb, WBC, differential, platelets); alkaline phosphatase, SGOT, electrolytes, creatinine, glucose, urinalysis (34.79); sputum cytology (15.71 × 2 = 31.42); chest radiograph (30.95); follow-up visit (26.40) | 172 |
| | Specialist consultant fee (102.45); chest radiograph (30.95 × 2 = 61.90); haematology, alkaline phosphatase, SGOT, electrolytes, creatinine, urinalysis, glucose (34.79 × 2 = 69.58); pulmonary function tests (30.20 + 31.50 = 61.70); sputum cytology (15.71 × 2 = 31.42) | 327 |
| Pre-op work-up | Bronchoscopy (102.10); laboratory charges (13.72); interpretation of biopsy (44.95); cytology of bronchial washings (6.86 + 15.90 = 22.76) | 184 |
| | Surgical consultation (52.80); ECG (15.35); arterial blood gases (19.40); CT scan (thorax) (60.20 × 50% = 30.10)[b] Mediastinoscopy with bronchoscopy (432.51) | 551 |
| Surgical procedure | Lobectomy to 85% (1 333.13) Pneumonectomy to 12% (1 413.41) (weighted average = 1 341.56) Limited resection to 3% (1 292.99) | 1 342 |
| Radiotherapy | Radiation consultation (102.45) Preradiotherapy haematology (31.36) Weekly haematology (3.92/week × 5 = 19.60) Radiation therapy (145/treatment × 25 treatments = 3 625) | 3 779 |
| Hospitalisation costs | Hospital costs for diagnosis, surgery and post-operative recovery (545.19 per day × 20 days = 10 903.80) | 10 904 |
| Follow-up costs in first year[a] | General assessment (52.40); general reassessment (37.70 × 2 = 75.40); partial assessment (22.40); chest radiograph (30.95 × 4 = 123.80); CBC (3.92 × 4 = 15.68); SMA-6 (17.64 × 4 = 70.56); alkaline phosphatase (4.90 × 4 = 19.60) clinic costs for 1 year (nursing support, overhead) (53.11 per visit × 17.5% (CPI) = 62.41 × four visits = 249.64) Total = 629.48 | 630 |
| | Total | 17 889 |

The costing model assumes that the diagnostic module includes the initial medical contact, diagnostic work-up tests and a bronchoscopy with biopsy. [a]Assume annual follow-up costs after first year = $630. [b]CT scan (thorax) added to 50% of patients only.

**Figure 1** Treatment schema for stage I and II non-small-cell lung cancer. [a]Ninety per cent of stage I and 85% of stage II receive surgical resection. [b]Ninety per cent of stage I and 85% of stage II receive complete resection.

each arborisation of the treatment algorithm. Similarly, a questionnaire survey was sent to the 163 radiation oncologists in Canada who are listed in the Canadian Association of Radiation Oncologists (CARO) membership directory. Forty-eight responses (29.4%) were obtained and the average of their responses was used to estimate the proportion of patients with a particular stage of lung cancer who would receive radiotherapy, as well as the dose and number of radiation treatments (fractions).

Patients who undergo a complete surgical resection (estimated by surgeons at 90% of stage I and 85% of stage II) are generally followed up without additional treatment. Twenty percent are assigned post-operative radical radiotherapy (54 Gy in 25 fractions) in the model, based on the questionnaire responses. Patients who have an incomplete surgical resection (estimated as 10% of stage I and 15% of stage II) are assigned post-operative radiotherapy in the model. Again, based on the questionnaire responses, these patients are assigned radical radiotherapy (54 Gy in 25 fractions). It is assumed that 10% of stage I and 15% of stage II patients are either medically unsuitable for surgical therapy or refuse surgical resection and that they would be treated by radical radiotherapy (54 Gy in 25 fractions).

Based on the advice of several academic thoracic surgeons, we assumed that follow-up in the first year would occur at 3 month intervals and include a physical examination, a chest radiograph and blood work (complete blood count, SMA-6 and alkaline phosphatase).

As an example of the detailed cost information included in each diagnostic and treatment algorithm, the cost components for one arm of stages I and II NSCLC are presented in Table I. Detailed cost breakdowns for the remaining treatment algorithms are not included in this document but are available upon request.

*Stage III and IV* The diagnostic and treatment algorithm for the management of patients with stage III or stage IV NSCLC is shown in Figure 2. Patients with stage IIIa and stage IIIb NSCLC are assumed not to be candidates for surgical resection, even though patients with single-station mediastinal nodal involvement (N2) commonly undergo surgical resection, as do those with T3, N0 or N1 lesions. Such cases make up only a small percentage (6%) of all surgically resected lung cancer (Holmes, 1989). It is also assumed that only stage IIIa patients require mediastinoscopy for staging. Responses from the questionnaire survey of radiation oncologists indicated that they would treat 85% of the patients with stage IIIa disease with radiotherapy to a moderately high dose (45 Gy in 20 fractions). A cervical mediastinoscopy is not included in the model for stage IIIb NSCLC. The dose of radiation for stage IIIb patients, 80% of whom would be offered treatment, is less (35 Gy in ten fractions), reflecting the general tendency to treat these patients less aggressively. Those patients not radiated receive no active treatment in the model. The frequency of follow-up assessments and the tests performed for stage IIIa patients after receiving radiotherapy are assumed to be similar to those assigned to the radiotherapy arms of stages I and II. The number of follow-up visits for all other stage III patients is fewer because of the short (9 months) median survival of these patients.

For patients with stage IV disease, the model assumes that standard treatment is best supportive care, consisting of analgesics and antibiotics. These individuals would undergo 10 days of diagnostic assessment, including a bone scan and a liver ultrasound to stage their disease. It is assumed that they would incur terminal care costs, consisting of palliative radiotherapy and hospitalisation, as described below.

*Determination of relapse and management of terminal care (NSCLC)* Patients with localised NSCLC who relapse after surgery and/or radiotherapy were assigned 2 days of hospitalisation for diagnostic work-up, as Canadian physicians frequently use this approach to expedite the diagnosis of relapse. Most of the work-up is done through ambulatory clinic visits. Investigations apart from blood work included a bone scan, CT scan and an abdominal ultrasound scan to identify sites of relapse. It is assumed that those who relapse and those who are diagnosed with lung cancer in the final stage (stage IV) would receive terminal care, which would include palliative radiotherapy, 23.6 days of hospitalisation and clinic visits, similar to the supportive care patients managed on BR-5. Palliative radiotherapy is used to relieve either local symptoms, such as airway obstruction or haemoptysis, or symptoms of distant metastatic disease, such as painful bone metastases or neurological deficits from brain metastases. The average number of treatment fractions necessary to palliate patients was extracted from the BR-5 study of best supportive care in advanced NSCLC, as was the average length of terminal care hospitalisation, the total number of follow-up clinic visits and the amount of nursing support (Jaakkimainen et al., 1990).

### Therapeutic approaches for small-cell lung cancer

The diagnostic and treatment algorithm for the management of patients with limited or extensive SCLC is shown in

**Figure 2** Treatment schema for stage III and IV non-small-cell lung cancer. [a]Eighty-five per cent of stage IIIA and 80% of stage IIIB receive radiotherapy. [a]Eight-five per cent of stage IIIA and 80% of stage IIIB receive radiotherapy.

Figure 3. In the model, patients with SCLC are assigned the same initial diagnostic tests and assessments as NSCLC, but, in addition, it is assumed that they receive standard staging tests, including a bone scan, CT brain scan, abdominal scan by CT or ultrasound and a bone marrow aspirate and biopsy.

*Limited disease* For patients with limited stage SCLC, standard treatment is assumed to consist of six courses of systemic chemotherapy, locoregional radiotherapy and prophylactic cranial irradiation. The standard combination chemotherapy costed in the model was the three-drug regimen cyclophosphamide, doxorubicin (Adriamycin) and vincristine (CAV), alternating with the two-drug combination of etoposide and cisplatin. The drug doses and schedule were the same as those used by the NCIC in its randomised study (BR-4), which demonstrated the superiority of this alternating chemotherapy approach (Evans *et al.*, 1987). It is assumed that patients with limited disease receive locoregional (chest) radiation (40 Gy in 20 fractions), as they did in another NCIC treatment protocol (BR-3) (Feld *et al.*, 1987). Furthermore, the model estimates that 70% of patients receive prophylactic cranial irradiation (20 Gy in ten fractions), based on the fact that virtually all complete responders and most partial responders receive this treatment in Canada.

The frequency and cost of follow-up visits were extracted from the BR-4 data. After the first year, follow-up consists of six visits per annum, each of which includes a physical examination, standard blood work, a chest radiograph and clinic visit costs.

*Extensive disease* The chemotherapy treatment costed for extensive small-cell lung cancer was the same as for limited disease. The amount of radiotherapy used in the treatment of extensive disease was extracted from the NCIC BR-4 study results. It is estimated that only 30% of extensive stage patients receive prophylactic cranial irradiation. Finally, it is assumed that 5% of patients with extensive small-cell lung cancer are either too frail or have medical conditions that preclude treatment with standard chemotherapy. These patients are assigned 6 days of hospitalisation plus follow-up costs, which include outpatient assessments, blood work, chest radiographs and clinic visit costs. It is assumed that they then receive terminal care, as described below.

*Determination of relapse and management of terminal care (SCLC)* Estimates of the cost of diagnosing relapse for limited disease include blood work, a chest radiograph, a bone scan, an abdominal ultrasound, 2 days of hospitalisation and three clinic visits. A CT brain scan is assigned to half the SCLC patients because of the high frequency of relapse in the central nervous system (Feld *et al.*, 1984).

Relapsing limited disease patients are not assigned any further chemotherapy, but are assumed to incur terminal care costs, consisting of seven fractions of palliative radiotherapy based on the observed practice during the BR-4 trial (Evans *et al.*, 1987), as well as the cost of 26 days of hospitalisation, which was the average of the two arms of BR-4. Patients with extensive disease (those who relapse after primary treatment, as well as those who are assumed not to be candidates for any additional therapy) are assigned only the cost of 26 days of hospitalisation for terminal care.

### *Summary of costs by stage and cell type*

*NSCLC* The estimated cost of diagnosis and treatment for each stage and therapeutic approach of NSCLC is shown in Table II. It can be seen that the initial cost of diagnosis and surgical treatment for stage I and II (excluding relapse costs) was $14 110. The cost of combined modality therapy (surgery plus radiotherapy) for patients with stage I and II was $17 889. For those patients who were not surgical candidates, but who were treated with radical radiotherapy, the initial cost of diagnosis and treatment was estimated to be $12 474. The irradiation of patients with stage IIIa and stage IIIb disease was somewhat less costly, at $11 714 and $9 347 respectively. It was estimated that stage IV patients would incur an initial cost of $6 333 for their diagnosis. However, significant costs are incurred when NSCLC patients relapse and resources are used to diagnose relapse ($1 528) and to provide terminal care ($10 331).

*SCLC* Table III summarises the costs of diagnosis and treatment of patients with SCLC by stage and therapeutic modality. Combined modality therapy for limited disease

**Figure 3** Treatment schema for small-cell lung cancer. [a]Ninety-five per cent of extensive disease receive combined chemotherapy and radiotherapy. [b]Seventy per cent of limited disease and 30% of treated extensive disease receive prophylactic cranial irradiation.

Table II Costs of diagnosis and treatment (NSCLC)

| Tumour stage and treatment | Diagnostic tests | Pre-operative staging tests | Surgery | Radiotherapy | Hospitalisation and clinic | Follow-up first year | Total |
|---|---|---|---|---|---|---|---|
| Stage I + II | | | | | | | |
| Surgery alone | 683 | 551 | 1342 | – | 10904 | 630 | 14110 |
| Surgery + post-operative radiotherapy | 683 | 551 | 1342 | 3779 | 10904 | 630 | 17889 |
| Radiotherapy only | 683 | 870 | – | 3748 | 6543 | 630 | 12474 |
| Stage IIIa | | | | | | | |
| Radiotherapy | 683 | 835 | – | 3023 | 6543 | 630 | 11714 |
| No radiotherapy | 683 | 714 | – | 103[a] | 6543 | 330 | 8373 |
| Stage IIIb | | | | | | | |
| Radiotherapy | 683 | 230 | – | 1561 | 6543 | 330 | 9347 |
| No radiotherapy | 683 | – | – | 103[a] | 5452 | 330 | 6568 |
| Stage IV | | | | | | | |
| Supportive care | 683 | 198 | – | – | 5452 | – | 6333 |

[a]Additional radiation consultation to determine that radiotherapy will not be administered. Follow-up costs after the first year are assumed to be similar to those in the first year. Relapse costs (diagnostic tests and hospitalisation) are assigned to all stages except non-radiotherapy arms of stages IIIa and IIIb and stage IV and are estimated to be $1528. Terminal care costs of $10331 (palliative radiotherapy and hospitalisation) are assigned to patients in the year of death.

Table III Costs of diagnosis and treatment (SCLC)

| Tumour stage and treatment | Diagnostic tests | Staging tests | Radio-therapy | Chemo-therapy | Hospitalisation and clinic | Follow-up | Total |
|---|---|---|---|---|---|---|---|
| Limited disease | | | | | | | |
| Chemotherapy + Radiotherapy | 683 | 405 | 4 065 | 5 428 | 8 110 | [a] | 18 691 |
| Extensive Disease | | | | | | | |
| Chemotherapy + Radiotherapy | 683 | 405 | 1 592 | 3 618 | 7 027 | [a] | 13 335 |
| Palliative care | 683 | 405 | – | – | 3 272 | 379 | 4,739 |

[a]Follow-up costs in first year are included in clinic costs. Assume annual follow-up costs after the first year are $944. Assume the chemotherapy/radiotherapy arms of limited and extensive disease can relapse. Relapse costs (diagnostic tests and hospitalisation) are determined to be $1 590. Assume terminal care costs are assigned to patients in the year of death. Terminal care costs for limited disease (palliative radiotherapy and hospitalisation) are $10 544. For extensive disease (hospitalisation only) they are $9 387.

incurred the highest cost, at $18 691. The combined use of chemotherapy and radiotherapy does improve survival and offers a small but definite chance of long-term survival. The cost of chemotherapy and radiotherapy to palliate a patient with extensive disease was approximately $13 325. The initial diagnostic work-up for patients felt to be too frail for chemotherapy was estimated to cost $4 739. The cost to determine relapse was estimated to be $1 590. Limited disease SCLC patients who relapsed incurred the additional costs of $10 544 for palliative radiotherapy and terminal care. Extensive disease patients were assigned only the cost of 26 days of hospitalisation ($9 387).

### Discussion

The costing model that we have developed assumes that all patients with lung cancer are treated according to practice guidelines appropriate to cell type and stage of the disease. In reality, this is not the case. Data on the demographics of the Canadian lung cancer population demonstrate that this is a disease of the elderly (Statistics Canada, 1992). Seventy-five percent of the patients diagnosed in Canada in 1988 were older than 60 years of age. Previous studies have shown that elderly patients with cancer are less likely to receive the same kind of care as younger patients (Samet *et al.*, 1986; Chu *et al.*, 1987; Sillman *et al.*, 1989), particularly if they have NSCLC (Guadagnoli *et al.*, 1990). As lung cancer is a lifestyle disease, often associated with other serious medical conditions and a poor performance status, many clinicians do not recommend treatment unless there is potential for curative surgery. In addition, numerous areas of controversy surround the treatment of locally advanced, inoperable lung cancer (Durrant *et al.*, 1971; Payne, 1988).

The proportion of patients assigned treatment in the model was based on the questionnaire responses of thoracic and radiation oncologists. It is not known how closely the questionnaire responses conform to actual practice or what proportion of all patients with a particular stage of disease actually receive the standard therapy. It is certainly true that there is considerable variation in the radiotherapeutic approach to locally advanced NSCLC in Canada. Duncan *et al.* (1993) observed a bimodal distribution of radiation dose fractionation schedules in response to a questionnaire on Canadian radiation oncology practice in the palliation of inoperable lung cancer. Their questionnaire presented clinical scenarios similar to the questionnaire used to survey oncologists for this study. They observed, as did we, a low-dose group that selected either 20 Gy in five fractions or 30 Gy in ten fractions and a high-dose group that typically prescribed 60 Gy in 30 fractions. In their 1990 survey, 20% of radiation oncologists when presented with a clinical scenario of a symptomatic 59-year-old patient with a hilar mass and positive mediastinoscopy chose a radical treatment, (30 fractions over 6 weeks), but 56% recommended palliative treatment. The determinants of the dose fractionation schedule included the age of the radiation oncologist, the geographic region of the country, departmental policy, workload and available resources. Significant differences in treatment approaches have been observed in the treatment of inoperable lung cancer between Canada, the United States and Great Britain (Maher *et al.*, 1992). Controversy also surrounds the use of prophylactic cranial irradiation (PCI). In Canada, PCI is given routinely to limited disease SCLC patients who achieve a complete remission. Patients with both limited and extensive disease may also be offered PCI in an effort to reduce the chances of debilitating neurological symptoms from metastases. In the United States, PCI is used less commonly out of concern for post irradiation neurological syndromes and possible medico-legal action (Kristjansen, 1989).

In the model, we chose to omit chemotherapy costing from the management of stage IV disease, despite the fact that Canadian oncologists conducted the pioneering study that demonstrated that chemotherapy for stage IV disease does prolong survival (Rapp *et al.*, 1988). A related study made the counterintuitive observation that chemotherapy can be less costly to the health care system than best supportive care (Jaakkimainen *et al.*, 1990). Nonetheless, we made this decision because a survey of Canadian medical oncologists in early 1985 showed that only 16% of oncologists would opt for systemic therapy if they themselves had stage IV lung cancer (MacKillop *et al.*, 1987). Using the model, we have undertaken simulations of various chemotherapeutic approaches to stage IV disease which we will report separately.

It is almost certainly true that patients do not have equal access to care. Patients living in remote areas, aboriginal peoples and immigrants unfamiliar with the Canadian health care system may all have difficulty gaining access to standard care. Unfortunately, it is not possible in Canada to determine from any database the proportion of patients who actually receive treatment for any stage of lung cancer. For these reasons, estimates of the total first year costs for treatment may exceed the actual expenditures.

Although the costing for diagnostic tests was based on a knowledge of the usual tests required to make a diagnosis, in practice patients often present with symptom complexes that do not initially suggest a diagnosis of lung cancer. As a result, other investigations may be undertaken which, in retrospect, are not necessary for the diagnosis of lung cancer. In addition, tests are frequently duplicated, as patients move through the health care system from primary care physician to consultant, particularly within academic health science centres. In this respect, the estimates for diagnostic tests are undoubtedly low relative to the actual expenditures. However, the total amount expended on diagnostic tests is small and therefore unlikely, even if doubled, to constitute a very significant component of the total expenditure on lung cancer. As evidence of this, we did sensitivity analyses for the frequency of thoracic CT scanning assuming 50%, 75% and 100% usage. In the Canadian health care system, the operational costs are already absorbed in hospital operating budgets and reflected in the per diem rate. The only incremental cost is the professional interpretation fee. In the model, we assumed that 50% of patients were scanned. If 75% were scanned the total health care cost would only increase by $62 519. If 100% of patients had thoracic CT scans, the cost increase would be $125 039, a 0.5% increase in total health care cost.

There are several other areas where the model may have underestimated costs. It was assumed that radiation therapy was only administered to outpatients. However, some patients are too frail or debilitated to be treated on an ambulatory basis. In addition, some come from remote areas and are kept in cancer lodges or in hospital until their radiotherapy treatment has been completed. The proportion of radiotherapy patients hospitalised for primary therapy has not been determined, but this would be an important piece of information for future refinement of the model.

Another source of a downward bias in the cost estimates is the fact that the model generally assumes that diagnostic procedures and treatment are uncomplicated. In reality, diagnostic and therapeutic interventions can be associated with side-effects which may be serious and result in delayed hospital discharge or require outpatient assessment and/or readmission. For example, a surgical resection may be complicated by wound infection, bronchopleural fistula or empyema. Radiation therapy to the chest may result in oesophagitis, requiring hospitalisation for hydration and nutritional support. Chemotherapy may induce febrile neutropenia requiring hospitalisation, or nausea and vomiting requiring intravenous fluid and nutritional support.

The per diem rate for hospitalisation used for this study is probably another underestimate. We used the per diem rate for tertiary care hospitals obtained from Statistics Canada's 'Annual Return of Hospitals–Hospital Indicators', which, in 1988–89 was $545.19 (Statistics Canada, 1991). An estimate of hospital costs derived from raw utilisation data obtained from Sunnybrook Medical Centre in Toronto for 29 surgical patients in 1992 was $776.10 (M Moffat, unpublished results). Complications of therapy and co-morbidity as well as unnecessary test ordering may, in part, explain the higher cost per day of hospitalisation at Sunnybrook Medical Centre.

The fact that treatment-related complications are not generally considered in the NSCLC component of the model will result in an underestimate of the true cost of lung cancer management. The hospitalisation and clinic costs associated with chemotherapy for small-cell lung cancer were extracted directly from the NCIC BR-4 clinical trial and do include the costs of hospitalising patients for therapy-related complications. These costs are, therefore, more representative of the true costs of the management of SCLC.

Finally, the costs of procedures and physician assessments were based on the Ontario fee schedule, and the cost of hospitalisation for chemotherapy and supportive care was derived largely from Ontario data. The cost of a fraction of radiotherapy was also derived from a study done in a cancer treatment facility in Ontario. The costs for these various components of health care will vary somewhat from province to province. Nonetheless, it is unlikely that there are major differences in these costs between the provincial health care systems in Canada.

This research was conducted as part of a larger study, to provide information on various diseases for a comprehensive microsimulation model called POHEM (for Population Health Model), which was developed at Statistics Canada (Wolfson, 1992). POHEM was designed to simulate the health status of the Canadian population, by integrating data on risk factors, disease onset and progression, health care resource utilisation, direct medical care costs and health outcomes. POHEM currently models lung cancer, breast cancer, coronary heart disease, arthritis and dementia, and a number of diseases are under development.

Despite the various limitations outlined above, the lung cancer model has a level of sophistication which, we believe, provides a realistic 'baseline' estimate of the cost per case of treatment by stage and therapeutic modality. The results of this lung cancer study have been incorporated into the POHEM framework (Gentleman *et al.*, 1991). This means that POHEM now contains a database against which new therapeutic or preventive interventions can be evaluated. The development of models such as the lung cancer model, and their inclusion in the POHEM framework, allows for the analysis of the interplay of risk factors, disease states and costs. It provides a valuable tool to reflect the overall health status of Canadians and to analyse the impact of health policies on the Canadian population.

**Acknowledgements**
The authors wish to acknowledge and thank the many members of the multidisciplinary team responsible for the completion of this project: Ronna Egan, Christian Houle and Monica Tomiak, from the Health Analysis and Modeling Group of the Social and Economic Studies Division of Statistics Canada; Jane Gentleman, Leslie Gaudette and others from the Health Statistics Division (Statistics Canada) for the provision of advice and assistance, in addition to the analysis of cancer registry data; Sylvana Beaulieu and Pierre David from the Social Survey Methods Division of Statistics Canada; Dr Eric Holowaty and staff of the Ontario Cancer Registry; Linda Buske and staff at the Health Information Division of Health and Welfare Canada for assistance in evaluating and interpreting the cost components of the study; Drs Andreas Laupacis, Aslam Anis, George Wells and David Stewart from the Clinical Epidemiology Unit of the Ottawa Civic Hospital; thoracic surgeons and radiation oncologists who completed and returned the survey of practice questionnaire; Drs Glenwood Goss, Diane Logan and Libni Eapen from the Ottawa Regional Cancer Centre who served as the local lung cancer expert panel; Dorothy Pineau and Chris Allen for their administrative and secretarial assistance.

### References

CHU J, DIEHR P, FEIGEL P, GLAEFKE G, BEGG C, GLICKSMAN A AND FORD L. (1987). The effect of age on the care of women with breast cancer in community hospitals. *J. Gerontol.*, **42**, 185-190.

DUNCAN G, DUNCAN W AND MAHER EJ. (1993). Patterns of palliative radiotherapy in Canada. *Clin. Oncol.*, **5**, 92-97.

DURRANT KR, BERRY RJ, ELLIS F, RIDEHALGH FR, BLACK JM AND HAMILTON WS. (1971). Comparison of treatment policies in inoperable bronchial carcinoma. *Lancet*, **1**, 715-719.

EVANS WK, FELD R, MURRAY N, WILLAN A, COY P, OSOBA D, SHEPHERD FA, CLARK DA, LEVITT M AND MACDONALD A. (1987). Superiority of alternating non-cross-resistant chemotherapy in extensive small cell lung cancer. *Ann. Intern. Med.*, **107**, 451-458.

FELD R, EVANS WK, DEBOER G, QUIRT IC, SHEPHERD FA, YEOH JL, PRINGLE JF, PAYNE DG, HERMAN JG, CHAMBERLAIN D, BROWN TC, BAKER MA, MYERS R, BLACKSTEIN ME AND PRITCHARD KI. (1984). Combined modality induction therapy without maintainence chemotherapy for small cell carcinoma of the lung. *J. Clin. Oncol.*, **2**, 294-304.

FELD R, EVANS WK, COY P, HODSON I, MACDONALD S, OSOBA D, PAYNE D, SHELLEY W AND PATER JL. (1987). Canadian multicentre randomized trial comparing sequential and alternating administration of two non-cross-resistant chemotherapy combinations in patients with limited small-cell carcinoma of the lung. *J. Clin. Oncol.*, **5**, 1401-1409.

GENTLEMAN JF, WILL BP, WOLFSON M AND EVANS WK. (1991). Microsimulation of health data: simulation of incidence, progression and mortality for lung cancer. In: *1991 Proceedings of the Statistical Computing Section*. American Statistical Association, Atlanta, Georgia, pp. 60-69.

GOODWIN PJ, FELD R, EVANS WK AND PATER J. (1988). Cost-effectiveness of cancer chemotherapy: an economic evaluation of a randomized trial in small-cell lung cancer. *J. Clin. Oncol.*, **6**, 1537-1547.

GUADAGNOLI E, WEITBERG A, MOR V, SILLIMAN RA, GLICKSMAN AS AND CUMMINGS FJ. (1990). The influence of patient age on the diagnosis and the treatment of lung and colorectal cancer. *Arch. Intern. Med.*, **150**, 1485-1490.

HOLMES EC. (1989). Surgical adjuvant therapy of non-small-cell lung cancer. *J. Surg. Oncol.*, (Suppl. 1), 26-33.

JAAKKIMAINEN L, GOODWIN PJ, PATER J, WARDE PM, MURRAY N AND RAPP E. (1990). Counting the costs of chemotherapy in a National Cancer Institute of Canada randomized trial in non-small cell lung cancer. *J. Clin. Oncol.*, **8**, 1301-1309.

KRISTJANSEN PEG. (1989). The role of cranial irradiation in the management of patients with small cell lung cancer. *Lung Cancer*, **5**, 264-274.

MACKILLOP WJ, O'SULLIVAN B AND WARD GK. (1987). Non-small cell lung cancer: how oncologists want to be treated. *Int. J. Radiat. Oncol. Biol. Phys.*, **13**, 929-934.

MAHER EJ, COIA LR, DUNCAN GG AND LAWTON PA. (1992). Treatment strategies in advanced and metastatic cancer: differences in attitude between the USA, Canada and Europe. *Int. J. Radiat. Oncol. Biol. Phys.*, **23**, 239-244.

NATIONAL CANCER INSTITUTE OF CANADA. (1992). *Canadian Cancer Statistics 1992*. National Cancer Institute of Canada: Toronto.

NATIONAL CANCER INSTITUTE OF CANADA. (1994). *Canadian Cancer Statistics 1994*. National Cancer Institute of Canada: Toronto.

PAYNE DG. (1988). Non-small cell lung cancer: should unresectable stage III patients routinely receive high-dose radiation therapy? *J. Clin. Oncol.*, **6**, 552-558.

RAPP E, PATER JL, WILLAN A, CORMIER Y, MURRAY N, EVANS WK, HODSON I, CLARK DA, FELD R, ARNOLD AM, AYOUB JI, WILSON KS, LATREILLE J, WIERZBICKI RF AND HILL DP. (1988). Chemotherapy can prolong survival in patients with advanced non-small cell lung cancer. Report of a Canadian multicentre randomized trial. *J. Clin. Oncol.*, **6**, 633-641.

SAMET J, HUNT WC, KEY C, HUMBLE CG AND GOODWIN JS. (1986). Choice of cancer treatment varies with the age of the patient. *JAMA*, **255**, 3385-3390.

SHIELDS TW. (1992). Screening, diagnosis, and staging of non-small cell lung cancer and consideration of unusual primary tumours of the lungs. *Curr. Opin. Oncol.*, **4**, 299-307.

SILLIMAN R, GUADAGNOLI E, WEITBERG A AND MOR V. (1989). Age as a predictor of diagnostic and initial treatment intensity in newly diagnosed breast cancer patients. *J. Gerontol.*, **44**, M46-M50.

SKARIN AT. (1993). Respiratory tract and head and neck cancer. In: *Scientific American Medicine* Rubenstein E and Federman DD (eds) pp. 1-17. Scientific American.

STATISTICS CANADA. (1991). *Annual Return of Hospitals-Hospital Indicators (1988-89)*, Catalogue 83-233.

STATISTICS CANADA. (1992). *Cancer in Canada 1988*, Supplement No. 8 to Health Reports, Catalogue 82-003S8.

WODINSKY H AND JENKIN RDT. (1987). The cost of radiation treatment at an Ontario regional cancer centre. *Canad. Med. Assoc. J.*, **137**, 906-909.

WOLFSON MC. (1992). POHEM-A Framework for Understanding and Modeling the Health of Human Populations. In: *Bureau of the Census Proceedings of the 1992 Annual Research Conference*, Arlington, Virginia, pp. 261-282. US Dept of Commerce: Washington DC.